# Observational study of patients in Spain with amyotrophic lateral sclerosis: correlations between clinical status, quality of life, and dignity

**DOI:** 10.1186/s12904-017-0260-6

**Published:** 2017-12-19

**Authors:** Yolanda Martínez-Campo, Christian Homedes, Ana Lazaro, Raquel Alarcón, David Campo, Mariona Riera, Raúl Domínguez, Mónica Povedano, Carlos Casasnovas

**Affiliations:** 1 ALS Functional Unit, Neurology Department, Bellvitge University Hospital– Biomedical Research Institute of Bellvitge (IDIBELL), C/ Feixa LLarga SN, 08906 Barcelona, L’Hospitalet de Llobregat Spain; 2 Neuromuscular Unit, Neurology Department, Bellvitge University Hospital– Biomedical Research Institute of Bellvitge (IDIBELL), C/ Feixa LLarga SN, 08906 Barcelona, L’Hospitalet de Llobregat Spain; 3 Clinical Sciences Department, Faculty of Medicine, University of Barcelona, C/ Feixa LLarga SN, 08906 Barcelona, L’Hospitalet de Llobregat Spain

## Abstract

**Background:**

Amyotrophic lateral sclerosis (ALS) is an incurable neurodegenerative disease that dramatically affects patients’ quality of life (QoL) and dignity of life (DoL). We aimed to study the impact of ALS on QoL and DoL and how these evolve throughout the duration of the disease.

**Methods:**

First, we performed an observational, descriptive study of 43 patients with ALS recruited from the ALS unit at our center and compared them with 20 healthy age- and sex-matched controls. Second, we performed a prospective cohort study, following up 23 patients with ALS over 3 months. All participants completed questionnaires about their functional status, QoL, and DoL.

**Results:**

QoL and DoL were significantly worse in the ALS group than in controls (both *p* < 0.001). During the three-month follow-up in the ALS cohort, statistically significant declines were observed in clinical status and QoL. For clinical status, median scores on the ALS Functional Rating scale changed from 30.95 points at baseline to 27.24 points after 3 months (*p* = 0.0003). For QoL, median scores on the ALS Assessment Questionnaire changed from 124.19 points at baseline to 131.81 at 3 months (*p* = 0.0062). However, no significant differences were found between the DoL scores at baseline (48.14 points) and 3 months (45 points) (*p*-value = 0.12).

**Conclusions:**

ALS is a neurodegenerative disease that affects QoL and DoL alike. We found that clinical status and QoL both deteriorated in patients with ALS as the disease progressed, but that DoL was preserved. However, our findings are limited by small sample sizes. The preservation of DoL may be due to multiple factors, including the therapies provided by the ALS unit. These findings suggest that alongside QoL, DoL may be an important target in the management and care of ALS patients.

## Background

Amyotrophic lateral sclerosis (ALS) is a fatal disease characterized by progressive degeneration of motor neurons in the brain and spinal cord [1]. The loss of motor neurons leads to progressive weakness that mainly impairs voluntary motor function, including that associated with walking, swallowing, speaking, and/or breathing. ALS may also cause severe cognitive and behavioral impairment such as frontotemporal dementia [2].

In clinical practice, ALS can be classified as spinal, bulbar, or respiratory depending on the location of the motor neurons affected at the onset of the disease. Patients with spinal onset typically develop problems with upper or lower limb mobility in the form of muscle weakness and cramps. In patients with bulbar onset, the initial symptoms are usually dysphagia and/or dysarthria, while in patients with respiratory onset, the initial symptom is usually dyspnea or even acute respiratory failure [3, 4].

ALS typically affects people aged 40–70 years old, and most patients (around 60%) are men. The annual incidence among Europeans is 2–3 per 100,000 in the general population [4]. However, variations are found in different parts of the continent, possibly due to environmental and/or genetic factors [5]. Although the contribution of environmental factors remains unclear, major advances have been made in our understanding of the genetic causes of ALS [4]. For instance, a better understanding of the roles of disturbed RNA metabolism and pathological protein aggregation has given new hope that novel diagnostic and therapeutic approaches can be developed [5].

Older age, male sex, family history of ALS, and bulbar onset are consistently reported as markers of poor prognosis. By contrast, low rates of symptom progression, better psychosocial factors, and better respiratory function are all associated with better outcomes. The effect on survival of enteral nutrition by percutaneous endoscopic gastrostomy (PEG) is unclear, but it has been shown that bi-level positive airway pressure (BPAP) and riluzole therapy can slightly improve survival (the latter by 3–6 months) [5].

Unfortunately, there is no cure for ALS at present, and the disease is normally fatal within 20 to 48 months from onset, although 10%–20% of patients with ALS survive longer than 10 years. The cause of death is normally respiratory insufficiency or aspiration pneumonia [6]. Since it is a degenerative disease without either a biological marker or curative treatment, diagnosis is based on the exclusion of diseases with similar clinical findings by effective treatment, coupled with evidence of upper and lower motor neurons being affected at multiple levels. To facilitate diagnosis, a number of diagnostic criteria have been developed, with the El Escorial criteria being the most widely used [7]. Due to the continued lack of an effective treatment for ALS, therapy focuses on symptom control and palliative care, aiming to maintain quality of life (QoL) as high as possible [5].

The ALS Functional Rating (ALSFR) scale is commonly used to assess physical symptom progression [8]. This is a validated questionnaire-based scale that measures physical functioning while patients with ALS carry out activities of daily living. The scale components are grouped into four domains: gross motor tasks, fine motor tasks, bulbar functions, and respiratory function. The scale ranges from 0 to 40, where lower scores indicate poorer function.

The meaning of QoL is difficult to describe because it encompasses objective and subjective indicators, a broad range of life domains, and individual values. A QoL assessment should not apply externally derived norms without reference to individual differences, and should allow objective comparisons to be made between the situations of specific groups and what is considered normal [8]. Standard indicators of QoL are wealth, employment, residence, physical and mental health, education, recreation and leisure time, and social belonging [9].

Given that physical and mental health are major factors in the evaluation of QoL, healthcare professionals must take these into account when planning therapeutic interventions. However, specialists frequently focus on resolving a specific pathology with aggressive therapy that can actually worsen the patient’s QoL and global functionality over the medium or long term. QoL is severely and progressively affected in patients with ALS [9], and health professionals have a duty to preserve it whenever possible [10]. The ALS Assessment Questionnaire (ALSAQ40) can be used to measure the subjective wellbeing of patients with ALS [11, 12] across five domains (physical mobility, activities of daily living and independence, eating and drinking, communication, and emotional reactions) over the last 2 weeks. The maximum score is 200 points, with higher scores indicating lower QoL [12].

Human dignity can be described as the ability to exercise free will and choice. Consequently, the concept of dignity of life (DoL) is intimately associated with the principle of autonomy in biomedical ethics: that is, it is always the patient who decides and who has the final say [13, 14]. One of the first studies to explore dignity therapy in people with ALS indicated that the end of life experience could be enhanced by supporting the unique identity of a person and by helping family members during bereavement [15]. The Patient Dignity Inventory (PDI) is a useful instrument for measuring DoL [13, 14]. This novel screening tool assesses multiple sources of distress salient to patients with limited life expectancies on a scale from 25 to 125, with higher scores indicating greater distress. It is a reliable, validated measure that has been derived from empirical studies on dignity concerns among the terminally ill [16–18]. Moreover, the questionnaire has been validated in multiple languages and applied in palliative care settings and studies worldwide. One national study of patients with cancer indicated that the PDI readily helped identify dignity-related distress in 76% of cases in which clinicians were previously unaware of distress, thereby allowing timely and targeted therapeutic responses [18, 19].

In the first part of this two-step study, we aimed to determine how ALS affects QoL and DoL in comparison with healthy controls. In the second part, we prospectively studied how the clinical progression of ALS affects DoL and QoL. To the best of our knowledge, no study in Spain has explored whether patients from an ALS unit suffer a decline in QoL and DoL, nor the relation of these with functional deterioration due to the disease.

## Methods

### Study design

We included patients who underwent routine follow-up at our center between December 2015 and April 2016. The ALS unit at our center (UFELA) manages patients with ALS from the Catalonia region, but mainly sees patients from our hospital’s immediate catchment area containing approximately 1.3 million people. First, we conducted a cross-sectional observational study of patients with ALS and compared them with 20 healthy controls. These latter were selected from among the authors’ relatives (YMC, AL, CH, RA, and DC) to present similar age and sex distributions, and were not excluded if they had non-complicated high blood pressure, dyslipidemia, or diabetes. Second, we performed a three-month prospective observational study with a cohort of patients from the first part of the study, who were being treated and followed up at our center for diagnosed ALS. In total, three questionnaires were administered: the ALSFR, the ALSAQ40, and the PDI.

### Inclusion and exclusion criteria

All included patients signed and dated an informed consent form and all had definitive ALS diagnosed according to the El Escorial criteria [7]. The exclusion criteria were cognitive impairment and motor neuron diseases other than ALS, including primary lateral sclerosis, progressive muscular atrophy, progressive bulbar palsy, and pseudobulbar palsy.

### Assessment tools

Basic demographic and disease-specific data were obtained for each patient, including their sex, current age, age of onset, time from symptom onset to diagnosis, region of onset (spinal upper limbs, spinal lower limbs, bulbar, or respiratory), and the administration of symptomatic treatment (PEG or BPAP). A control group of 20 healthy volunteers was used for comparison. All participants (patients and controls) completed the three study questionnaires at baseline and after 3 months, and their progression was evaluated by the difference in scores.

### Statistical analysis

StataCorp Version 13.1 was used for statistical analysis. Percentages (sex, use of PEG or BPAP, clinical onset) and means (age at ALS onset and time for disease evolution) were used for descriptive analysis. Two-sample Student *t*-tests with unequal variances were used to compare the results of functional, QoL, and DoL parameters for the ALS and control groups. A *p*-value of ≤0.05 was taken as indicating statistical significance. In the prospective study, mean scores were calculated for the three questionnaires and the means obtained from the two interviews were compared individually.

## Results

### Observational study

**Fig. 1:**
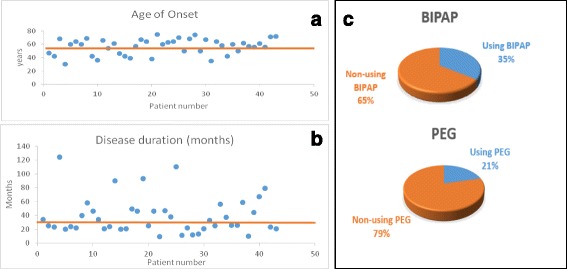
**a** Distribution of age of onset in the sample. **b** Distribution of disease duration. **c** Use of PEG and BPAP

We evaluated 43 patients with ALS, whose mean age was 60.33 years (Fig. 1a). Males predominated (53.5%), the mean age at ALS onset was 56.67 years (range, 30–75 years) (Fig. 1b), and the mean time for disease evolution was 39.16 months (range was between 9 and 124 months) (Fig. 1c). Nine patients (21%) required PEG, 15 (35%) required BPAP (Fig. 1c), and 4 required both. Physical status, patient functionality, and QoL were assessed with the ALSFR and ALSAQ40 scales, while dignity was assessed with the PDI scale. All patients responded to all the questionnaires. The median scores obtained on the ALSFR, ALSAQ40, and PDI scales were 30.76 points (range, 12–47), 119.30 points (range, 52–198), and 44.76 points (range, 28–66), respectively.

A significantly worse QoL was found in patients who required BPAP or PEG (*p* = 0.0092 and *P* = 0.0108, respectively). There were no significant differences in sex by age (*p* = 0.7658), age of ALS onset (*p* = 0.6600), clinical onset (*p* = 0.7059), duration of the disease (*p* = 0.6181), need for PEG (*p* = 0.5559) or BIPAP (*p* = 0.5405), ALSFR (*p* = 0.9219), QoL (*p* = 0.5626), or DoL (*p* = 0.4467). Neither were any significant differences observed with regard to clinical onset and duration of the disease (*p* = 0.1141), need for BIPAP (*p* = 0.6527), ALSFR (*p* = 0.4888), QoL (*p* = 0.5742), or DoL(*p* = 0.9753). Unsurprisingly, however, PEG was required more frequently in patients with bulbar/respiratory onset than in those with spinal onset (*p* = 0.0045). Similarly, no significant differences were found in QoL or DoL by sex (p = 0.5626 and 0.4467, respectively), age of ALS onset (*p* = 0.3982 and 0.3325), clinical onset (spinal, *p* = 0.060 and 0.187; bulbar, *p* = 0.126 and 0.995; respiratory, *p* = 0.340 and 0.058), or disease duration (*p* = 0.660 and 0.3982). There were no significant differences observed for PEG or DoL (*p* = 0.5750).

**Fig. 2:**
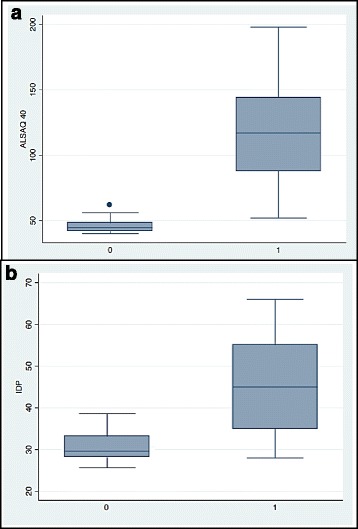
**a** Statistical differences in ALSAQ40 between ALS patients and the control cohort (*p* < 0.001). **b** Statistical differences in PDI between ALS patients and the control cohort (*p* < 0.001)

The average age of the control cohort was 61.9 years old, and males predominated (55%). The mean scores and standard deviations on the ALSAQ40 and PDI differed significantly (both *p* < 0.001) between the ALS and control cohorts (Fig. 2a and b).

### Prospective study

We followed up 23 patients with ALS over 3 months. Most patients responded to both questionnaires, but 2 (8.7%) died from ALS-related respiratory failure before finishing the study. There was a slight female predominance in the cohort (52.2%). The means for age, age of onset, and time for disease evolution were 58.9 years, 54.7 years, and 3.66 years, respectively. Onset was bulbar in 6 patients (26%), spinal in 15 (65.2%), and respiratory in 2 (8.7%). Among those with spinal onset, 11 (47.8%) were affected in the lower limbs first and 4 (17.4%) in the upper limbs first. Ten patients (43.5%) started BPAP and 6 (26%) started PEG over the three-month study period.

#### Clinical status

**Fig. 3:**
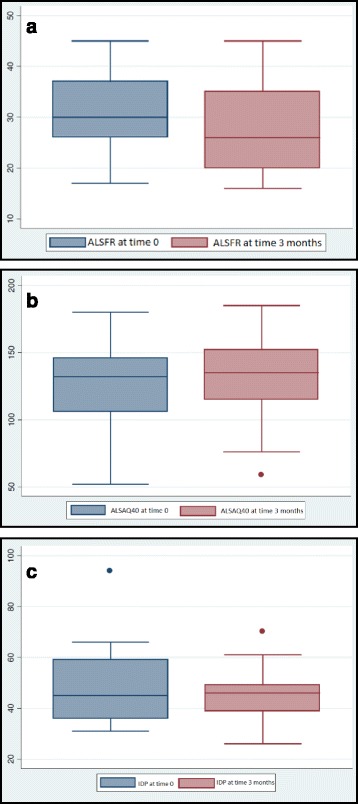
**a** Comparison of patients’ clinical status at time 0 and at 3 months. **b** Comparison of patients’ quality of life at time 0 and at 3 months. **c** Comparison of patients’ dignity of life at time 0 and at 3 months

Patients’ clinical status and functionality, evaluated by means of the ALSFR scale, showed significant worsening from baseline (ALSFR scale_1_) to 3 months (ALSFR scale_2_), with median scores of 30.95 and 27.24 points, respectively (*p* = 0.0003; Fig. 3a).

#### Quality of life

The ALSAQ40 scale showed significant worsening from baseline (ALSAQ40 scale_1_) to 3 months (ALSAQ40 scale_2_), with median scores of 124.19 and 131.81 points, respectively (*p* = 0.0062; Fig. 3b).

#### Dignity of life

DoL, evaluated by means of the PDI scale, improved slightly with disease progression, but no significant differences were found between the scores at baseline (PDI scale_1_) and 3 months (PDI scale_2_); the median scores were 48.14 and 45 points, respectively (*p* = 0.12; Fig. 3c).

## Discussion

All chronic diseases have major effects on QoL [7]. Patients with chronic obstructive pulmonary disease, asthma, osteoarthritis, and stroke have all been shown to be at increased risk of limitations to daily activities that are associated with lower scores on QoL questionnaires. One of the most disabling factors in these groups is depression [20, 21]. The QoL scores of patients with progressive chronic diseases also reportedly decrease with disease progression. Patients with type I diabetes, for example, have significantly lower QoL scores after 23.5 years’ follow-up as diabetes-related complications and psychiatric events develop [22], while patients with terminal cancer report significantly reduced QoL scores in the last months of life [23].

In patients with neurological disorders such as multiple sclerosis, there is a strong inverse correlation between disability level and QoL test scores, with a close relationship to depression and fatigue severity [21]. Similar patterns are seen in ALS, with specific correlations between increased physical dependency and depression, and between speech impairment and anxiety [24]. The irremediable progression of ALS tends to be reflected in a declining QoL. The goal for clinicians and caregivers is to slow this progression and render its impact less significant. These professionals must, therefore, focus interventions on symptom control and palliation, making decisions in the context of multidisciplinary teams [11].

Although there are multiple references to QoL in chronic progressive diseases [19], few studies mention DoL. However, dignity is an important concept that has recently emerged as an important outcome measure for progressive, degenerative conditions such as ALS, Huntington’s disease, or Parkinson’s disease. The inability of clinicians to alter disease progression significantly is sometimes taken to indicate resignation to the fact that nothing more can be done; but this is precisely why everything possible must be offered to provide appropriate psychological support that upholds patient dignity at all times [25, 26].

The epidemiological features of the patients in the observational part of this study were comparable to previously reported studies; hence, we believe that our sample was representative of patients with ALS. Unsurprisingly, patients reported worsening physical function, QoL, and DoL in comparison with the healthy controls. This deterioration did not seem to be associated with clinical or epidemiological factors (onset, age of onset, or sex), but in contrast with other studies [27, 28], the need to use technical aids such as BPAP and PEG had a significant and negative association with the QoL of ALS patients. We consider that the declines in QoL in these patients reflected disease progression more than the need to use the technical aid itself.

In the prospective study, after 3 months’ follow-up, statistically significant declines were observed in both physical function and QoL (measured by the ALSFR and ALSAQ40 scales, respectively), comparable to results in other studies of QoL in patients with ALS [25, 26]. All the patients presented a certain degree of functional deterioration with a mean worsening of 3.7 points on ALSFR. ALSFR ranges from 0 to 40, so a drop of 3.7 points represents a significant functional impact on the patients. Furthermore, a drop of 6 points on the ALSAQ40 scale (which ranges from 0 to 200) shows a clear tendency toward worsening QoL, which although slight, was already statistically significant in just 3 months of follow-up. However, despite significant deteriorations in these domains as the disease progressed, patients did not show a decline in DoL, as measured by the PDI. In fact, a slight non-significant increase in DoL was reported. A possible explanation is that our center is staffed by skilled nurses, psychologists, and caseworkers who provide psychological support, prepare for future disease progression, and encourage communication about end-of-life issues. These factors have each been associated with higher DoL scores, independently of disease progression [26]. Interdisciplinary care is also known to be a significant factor in the maintenance of dignity in patients with ALS [11], probably due to the sense of security that is instilled by receiving care from multiple specialists.

Unfortunately, there are limitations to this research. Notably, the numbers of patients in the first (46) and second [23] parts of the study, as well as the length of follow-up time (3 months), were low, limiting the statistical power of the study. However, this study included a prospective component in which the number of patients and follow-up period were sufficient to show statistically significant changes. A future prospective study should be designed with more patients followed over a longer period to corroborate our results. We also chose not to recruit relatives of ALS patients as controls to avoid the potential bias of caregiver burnout. Instead, the healthy control cohort was selected from among the relatives of the study authors, matching by age and sex distribution. Despite the valid justification, this could represent another limitation.

Due to the considerable physical and emotional repercussions of ALS, treatment should seek to improve QoL and DoL in all patients [15, 23–25]. It is important that we do not focus on traditional, disease-specific therapy, but instead think about more general and contemporary management principles. We believe that therapy focusing on DoL and QoL may prove useful in many progressive neurodegenerative conditions and other lethal diseases that have no treatment [15, 25, 26].

## Conclusions

ALS is a neurodegenerative disease that clearly affects QoL and dignity. In this pilot study, we identified significant deteriorations in clinical status, functionality, and QoL, but not in DoL, as the disease progressed. It is likely that this preservation of dignity is multifactorial in nature, being closely related to the use of therapies derived from a multidisciplinary approach to the disease. We believe that DoL, not solely QoL, could be an important target in the management of ALS.

## Abbreviations

ALS: Amyotrophic Lateral Sclerosis
ALSAQ40: Amyotrophic Lateral Sclerosis Assessment Questionnaire
ALSFR: ALS Functional Rating
BIPAP: Bi-level positive airway pressure
DoL: Dignity of Life
MNs: Motor neurons
PEG: Percutaneous endoscopic gastrostomy
QoL: Quality of Life
UFELA: ALS Unit in the Bellvitge University Hospital
